# DNA barcoding of *Deltocephalus* Burmeister leafhoppers (Cicadellidae, Deltocephalinae, Deltocephalini) in China

**DOI:** 10.3897/zookeys.867.35058

**Published:** 2019-07-29

**Authors:** Hong Zhang, Yalin Zhang, Yani Duan

**Affiliations:** 1 School of Plant Protection, Anhui Agricultural University, Hefei, Anhui Province 230036, China Anhui Agricultural University Hefei China; 2 Key Laboratory of Plant Protection Resources and Pest Management of the Ministry of Education, Entomological Museum, Northwest A&F University, Yangling, Shaanxi Province 712100, China Northwest A&F University Yangling China

**Keywords:** COI, genetic distance, morphological variant

## Abstract

We investigated the feasibility of using the DNA barcode region in identifying *Deltocephalus* from China. Sequences of the barcode region of the mitochondrial COI gene were obtained for 98 specimens (*Deltocephalus vulgaris* – 88, *Deltocephalus pulicaris* – 5, *Deltocephalus uncinatus* – 5). The average genetic distances among morphological and geographical groups of *D. vulgaris* ranged from 0.9% to 6.3% and among the three species of *Deltocephalus* ranged from 16.4% to 21.9% without overlap, which effectively reveals the existence of a “DNA barcoding gap”. It is important to assess the status of these morphological variants and explore the genetic variation among Chinese populations of *D. vulgaris* because the status of this species has led to taxonomic confusion because specimens representing two distinct morphological variants based on the form of the aedeagus are often encountered at a single locality. Forty-five haplotypes (*D. vulgaris* – 36, *D. pulicaris* – 5, *D. uncinatus* – 4) were defined to perform the phylogenetic analyses; they revealed no distinct lineages corresponding either to the two morphotypes of *D. vulgaris* or to geographical populations. Thus, there is no evidence that these variants represent genetically distinct species.

## Introduction

China contains threatened biodiversity hotspots, including one spanning the Palearctic and Oriental regions and containing a high level of species diversity (Lin et al. 2010). In these regions, accurate identification of extant species is of great significance, although the taxonomic expertise is limited. Traditionally, identification of most species has been based on morphology. However, the availability of inexpensive DNA sequencing technology now provides additional tools not only for routine species identification but also for testing the validity of morphology-based species concepts. DNA barcoding is a simple, effective tool, that can identify and delimit species, including some complex taxa, rapidly and accurately using a standard short DNA sequence of the mitochondrial cytochrome c oxidase I (COI) (Hebert et al. 2003, 2004b; Ward et al. 2005; Hajibabaei et al. 2006). This method has been widely recognized and accepted in molecular phylogenetic studies (Hebert et al. 2003). The COI-based identification system has achieved remarkable success discriminating species across numerous animal groups, including birds (Hebert et al. 2004b), fishes (Hubert et al. 2008), and the insect orders Lepidoptera (Hebert et al. 2004a; Hajibabaei et al. 2006; Yang et al. 2012; Ashfaq et al. 2013), Ephemeroptera (Ball et al. 2005), and Hymenoptera (Smith et al. 2008). But this technology has also failed to identify species accurately under certain circumstances. For example, in a study of 449 species of Diptera and using 1333 COI sequences, Meier et al. (2006) obtained an identification success rate below 70% due to extensive overlap in inter- and intraspecific genetic distances. Within the dipteran family Calliphoridae, Whitworth et al. (2007) found that only 60% of species tested could be identified reliably.

Deltocephalini feed on grasses and sedges and are diverse and abundant in grassland ecosystems. This group contains 73 genera and 613 species around the world. *Deltocephalus*, type genus of this tribe contains 62 species distributed in the Old World and New World. Some species of this genus can transmit pathogenic diseases to economically important plants and are important economic pests; therefore, tools are needed for their rapid and accurate identification. Four species are described from China, two of them transmit pathogenic diseases. Identification of leafhopper species in most genera now requires dissection and examination of the male genitalia. However, some taxonomically problematic species apparently exhibit substantial intraspecific variation in male genital structures, and this causes confusion among taxonomists. One such practical example is *D. vulgaris*, which has well-documented morphological differences in the shape of the aedeagus (Figs 2, 3). Dash and Viraktamath (1998) first reported morphological variation in this species when they reviewed the genus *Deltocephalus* from India. Webb and Viraktamath (2009) also reported two forms of the aedeagus despite many shared morphological features in the species. Zhang and Duan (2011) redescribed *D. vulgaris* with detailed drawings and photos, illustrating these obvious morphological differences.

Based on DNA barcoding of leafhoppers, 63 barcodes from 45 species in Japan (15 subfamilies and 37 genera without Deltocephalini) were analysed (Kamitani 2011). DNA barcodes from 546 adult specimens of leafhoppers, planthoppers and treehoppers (Hemiptera, Auchenorrhyncha) were obtained from Barrow Island and analysed (Gopurenko et al. 2013). Species determination of members in the genus *Aphrodes* (Hemiptera, Cicadellidae) based on vibrational signals, mitochondrial DNA and morphology were performed (Bluemel et al. 2014). A total of 1482 specimens based on DNA barcodes of Nearctic Auchenorrhyncha (Insecta, Hemiptera) were studied by Foottit et al. (2014). The boundaries of seven closely related species of the evacanthine leafhopper genus *Bundera* (Cicadellidae, Evacanthinae) based on DNA barcoding, morphology and hyperspectral reflectance profiling was investigated by Wang et al. (2016), and a revision of the genus *Orosius* (Cicadellidae, Deltocephalinae, Opsiini) based on morphological and DNA barcoding was undertaken by Fletcher et al. (2017). Although, DNA barcoding research has been applied to these groups of leafhoppers, until now, a few molecular data are available for *Deltocephalus*. Therefore, a better understanding of *Deltocephalus*, and particularly the variation of *D. vulgaris* based on molecular data, is urgently needed.

In this study, we studied 98 COI sequences from three species of *Deltocephalus*. DNA barcoding data were used to investigate genetic variation of Chinese populations of *D. vulgaris* and to determine whether the morphological variants previously identified in this species represent distinct lineages. Our specific aims were to test the feasibility of using DNA barcoding data for identification of species of *Deltocephalus*, to determine the levels of the genetic variation within *D. vulgaris*, and to preliminarily discuss its possible correlation with morphological variation and biogeographic patterns.

## Material and methods

### Taxon sampling

**Figure 1.**
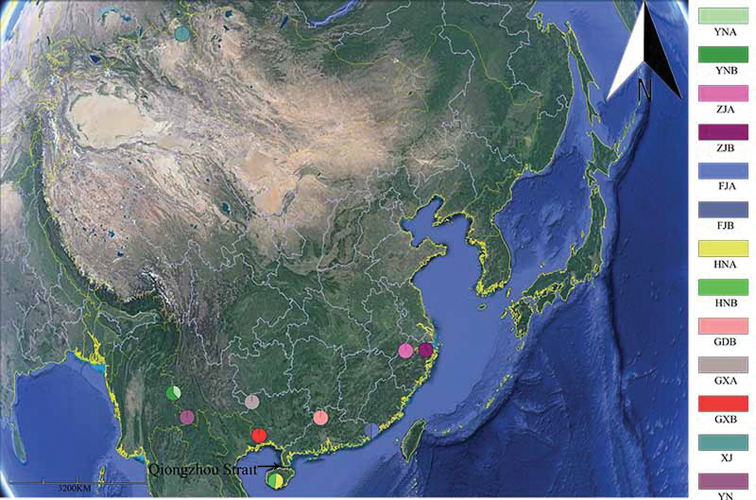
Distribution of *Deltocephalus* in China, codes same as in Table 1.

**Figure 2.**
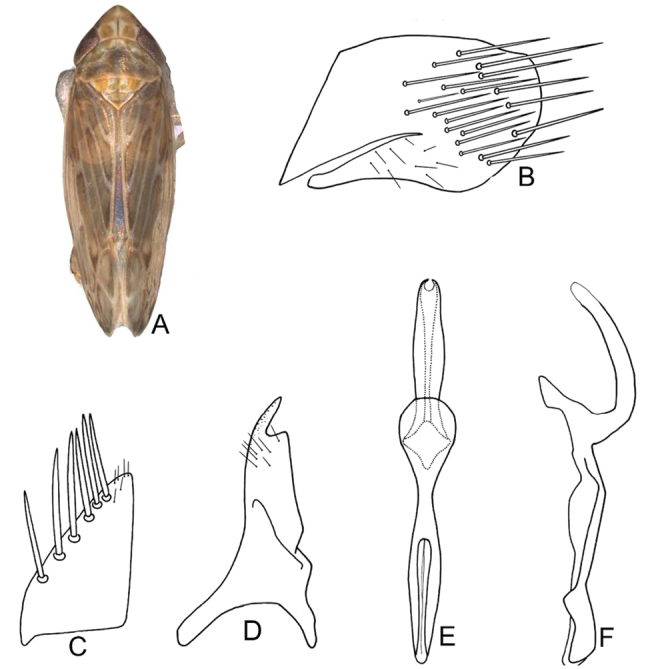
Morphological variant marked with A for *D. vulgaris***A** habitus in dorsal view **B** subgenital plate **C** subgenital plate **D** style **E** aedeagus and connective, dorsal view **F** aedeagus and connective, lateral view (after Zhang and Duan 2011).

**Figure 3.**
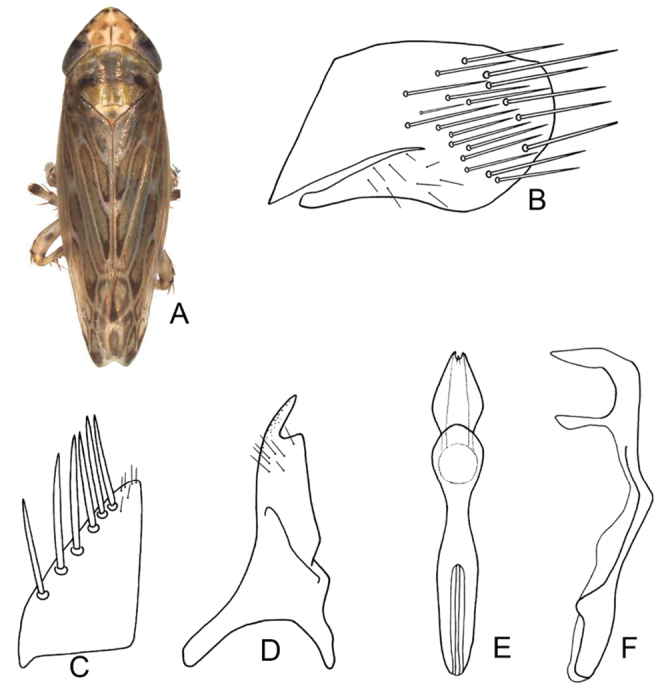
Morphological variant marked with B for *D. vulgaris***A** habitus in dorsal view **B** subgenital plate **C** subgenital plate **D** style **E** aedeagus and connective, dorsal view **F** aedeagus and connective, lateral view (after Zhang and Duan 2011).

A total of 98 specimens of *Deltocephalus* (*D. vulgaris* – 88, *D. pulicaris* – 5, *D. uncinatus* – 5) were collected with an insect sweep net in the daytime and by a light trap at night. Specimens were all collected directly into 95% or 100% ethanol and stored in -80 °C prior to study. The sample included *D. vulgaris*, *D. uncinatus* and *D. pulicaris* to facilitate comparison of inter- to intraspecific genetic variation in this group. *Deltocephalus vulgaris* specimens were divided into 11 groups based on their morphological differences and different geographical distributions in China (Table 1, Figs 1–3). Voucher specimens were deposited in the Key Laboratory of Plant Protection Resources and Pest Management of Ministry of Education, Entomological Museum, Northwest A&F University, Yangling, Shaanxi Province, China (NWAFU) and the School of Plant Protection, Anhui Agricultural University, Hefei, Anhui Province, China (AAU).

**Table 1.** List of samples studied and their relevant information.

Species	Group code	Sample size	Individual code	Haplotype	Locality	GenBank accession
* D. vulgaris *	YNA	8	YNA1	Hap1	Banhong Town, Yunnan Province	MK764780
			YNA2	Hap2	Banhong Town, Yunnan Province	MK764781
			YNA3	Hap3	Banhong Town, Yunnan Province	MK764782
			YNA4	Hap2	Banhong Town, Yunnan Province	MK764783
			YNA5	Hap4	Banhong Town, Yunnan Province	MK764784
			YNA6	Hap2	Banhong Town, Yunnan Province	MK767485
			YNA7	Hap1	Banhong Town, Yunnan Province	MK764786
			YNA8	Hap2	Banhong Town, Yunnan Province	MK764787
	YNB	13	YNB1	Hap5	Banhong Town, Yunnan Province	MK764788
			YNB2	Hap1	Banhong Town, Yunnan Province	MK764789
			YNB3	Hap5	Banhong Town, Yunnan Province	MK764790
			YNB4	Hap5	Banhong Town, Yunnan Province	MK764791
			YNB5	Hap5	Banhong Town, Yunnan Province	MK764792
			YNB6	Hap5	Banhong Town, Yunnan Province	MK764793
			YNB7	Hap5	Banhong Town, Yunnan Province	MK764794
			YNB8	Hap6	Banhong Town, Yunnan Province	MK764795
			YNB9	Hap7	Banhong Town, Yunnan Province	MK764796
			YNB10	Hap8	Banhong Town, Yunnan Province	MK764797
			YNB11	Hap5	Banhong Town, Yunnan Province	MK764798
			YNB12	Hap5	Banhong Town, Yunnan Province	MK764799
			YNB13	Hap5	Banhong Town, Yunnan Province	MK764800
	ZJA	7	ZJA1	Hap9	Lin’an County, Zhejiang Province	MK764801
			ZJA2	Hap10	Lin’an County, Zhejiang Province	MK764802
			ZJA3	Hap11	Lin’an County, Zhejiang Province	MK764803
			ZJA4	Hap12	Lin’an County, Zhejiang Province	MK764804
			ZJA5	Hap13	Lin’an County, Zhejiang Province	MK764805
			ZJA6	Hap12	Lin’an County, Zhejiang Province	MK764806
			ZJA7	Hap12	Lin’an County, Zhejiang Province	MK764807
	ZJB	8	ZJB1	Hap14	Kowloon Mountain, Zhejiang Province	MK764808
			ZJB2	Hap10	Kowloon Mountain, Zhejiang Province	MK764809
			ZJB3	Hap15	Kowloon Mountain, Zhejiang Province	MK764810
			ZJB4	Hap12	Kowloon Mountain, Zhejiang Province	MK764811
			ZJB5	Hap16	Kowloon Mountain, Zhejiang Province	MK764812
			ZJB6	Hap17	Kowloon Mountain, Zhejiang Province	MK764813
			ZJB7	Hap18	Kowloon Mountain, Zhejiang Province	MK764814
			ZJB8	Hap19	Kowloon Mountain, Zhejiang Province	MK764815
	FJA	7	FJA1	Hap20	Shajian Town, Fujian Province	MK764816
			FJA2	Hap20	Shajian Town, Fujian Province	MK764817
			FJA3	Hap5	Shajian Town, Fujian Province	MK764818
			FJA4	Hap21	Shajian Town, Fujian Province	MK764819
			FJA5	Hap5	Shajian Town, Fujian Province	MK764820
			FJA6	Hap20	Shajian Town, Fujian Province	MK764821
			FJA7	Hap5	Shajian Town, Fujian Province	MK764822
	FJB	7	FJB1	Hap22	Shajian Town, Fujian Province	MK764823
			FJB2	Hap20	Shajian Town, Fujian Province	MK764824
			FJB3	Hap20	Shajian Town, Fujian Province	MK764825
			FJB4	Hap20	Shajian Town, Fujian Province	MK764826
			FJB5	Hap20	Shajian Town, Fujian Province	MK764827
			FJB6	Hap8	Shajian Town, Fujian Province	MK764828
			FJB7	Hap23	Shajian Town, Fujian Province	MK764829
* D. vulgaris *	HNA	9	HNA1	Hap24	Jianfeng Mountain, Hainan Province	MK764830
			HNA2	Hap8	Jianfeng Mountain, Hainan Province	MK764831
			HNA3	Hap8	Jianfeng Mountain, Hainan Province	MK764832
			HNA4	Hap8	Jianfeng Mountain, Hainan Province	MK764833
			HNA5	Hap8	Jianfeng Mountain, Hainan Province	MK764834
			HNA6	Hap25	Jianfeng Mountain, Hainan Province	MK764835
			HNA7	Hap8	Jianfeng Mountain, Hainan Province	MK764836
			HNA8	Hap26	Jianfeng Mountain, Hainan Province	MK764837
			HNA9	Hap27	Jianfeng Mountain, Hainan Province	MK764838
	HNB	8	HNB1	Hap20	Jianfeng Mountain, Hainan Province	MK764839
			HNB2	Hap28	Jianfeng Mountain, Hainan Province	MK764840
			HNB3	Hap29	Jianfeng Mountain, Hainan Province	MK764841
			HNB4	Hap30	Jianfeng Mountain, Hainan Province	MK764842
			HNB5	Hap8	Jianfeng Mountain, Hainan Province	MK764843
			HNB6	Hap31	Jianfeng Mountain, Hainan Province	MK764844
			HNB7	Hap8	Jianfeng Mountain, Hainan Province	MK764845
			HNB8	Hap8	Jianfeng Mountain, Hainan Province	MK764846
	GDB	9	GDB1	Hap32	Patio Hill, Guangdong Province	MK764847
			GDB2	Hap8	Patio Hill, Guangdong Province	MK764848
			GDB3	Hap8	Patio Hill, Guangdong Province	MK764849
			GDB4	Hap8	Patio Hill, Guangdong Province	MK764850
			GDB5	Hap8	Patio Hill, Guangdong Province	MK764851
			GDB6	Hap20	Patio Hill, Guangdong Province	MK764852
			GDB7	Hap8	Patio Hill, Guangdong Province	MK764853
			GDB8	Hap8	Patio Hill, Guangdong Province	MK764854
			GDB9	Hap8	Patio Hill, Guangdong Province	MK764855
	GXA	4	GXA1	Hap33	Lingyun County, Guangxi Province	MK764856
			GXA2	Hap1	Lingyun County, Guangxi Province	MK764857
			GXA3	Hap34	Lingyun County, Guangxi Province	MK764858
			GXA4	Hap20	Lingyun County, Guangxi Province	MK764859
	GXB	8	GXB1	Hap35	Shangsi County, Guangxi Province	MK764860
			GXB2	Hap20	Shangsi County, Guangxi Province	MK764861
			GXB3	Hap32	Shangsi County, Guangxi Province	MK764862
			GXB4	Hap1	Shangsi County, Guangxi Province	MK764863
			GXB5	Hap5	Shangsi County, Guangxi Province	MK764864
			GXB6	Hap5	Shangsi County, Guangxi Province	MK648065
			GXB7	Hap20	Shangsi County, Guangxi Province	MK764866
			GXB8	Hap36	Shangsi County, Guangxi Province	MK764867
* D. pulicaris *	XJ	5	XJ1	Hap37	Altay City, Xinjiang Province	MK764868
			XJ2	Hap38	Altay City, Xinjiang Province	MK764869
			XJ3	Hap39	Altay City, Xinjiang Province	MK764870
			XJ4	Hap40	Altay City, Xinjiang Province	MK764871
			XJ5	Hap41	Altay City, Xinjiang Province	MK764872
* D. uncinatus *	YN	5	YN1	Hap42	Menglong Town, Yunnan Province	MK764873
			YN2	Hap43	Menglong Town, Yunnan Province	MK764874
			YN3	Hap43	Menglong Town, Yunnan Province	MK764875
			YN4	Hap44	Menglong Town, Yunnan Province	MK764876
			YN5	Hap45	Menglong Town, Yunnan Province	MK764877

Note: individual code with province initials and A or B and number; A and B are representative of two different morphological variants of *D. vulgaris* respectively.

### Morphology

Morphological observations were made using an Olympus SZX10 stereoscopic microscope (Olympus Corporation, Tokyo, Japan). All photographs and drawings were modified with Adobe Photoshop CS.

### DNA extraction, amplification and sequencing

Total genomic DNA was extracted from the whole abdomen of each leafhopper using the EasyPure Genomic DNA Kit (EE101; Transgen, Beijing, China) following the manufacturer’s instructions with the following modifications: abdomen incubated at 55 °C for about 20 hours, and with a nondestructive DNA extraction procedure to allow subsequent morphological observation. Genomic DNA extracts were stored in a freezer at -20 °C.

The barcode region (630bp) of the COI gene was amplified using primer combination (Folmer et al. 1994), LCO1490 (5’–GGT CAA ATC ATA AAG ATA TTG G–3’) and HCO2198 (5’–TAA ACT TCA GGG TGA CCA AAA AAT CA–3’) by the standard polymerase chain reaction (PCR) method. Total reaction volume was 25 μl, containing 12.5 μl of 2×Taq MasterMix, 8.5 μl of double distilled water (ddH_2_O), 2 μl of forward and reverse primer (1 μl, respectively), and 2 μl of DNA template solution. The following thermal cycling protocol was used: an initial denaturation step at 94 °C for 3 min, followed by 5 cycles of denaturation at 94 °C for 1 min, annealing at 45 °C for 1.5 min and extension at 72 °C for 1.5 min, followed by 35 cycles of denaturation at 94 °C for 1 min, annealing at 53.5 °C for 1 min and extension at 72 °C for 1 min, with a final extension of at 72 °C for 5 min, and ending with incubation at 12 °C.

The PCR products were examined using 1% agarose gel electrophoresis with ethidium bromide stain to check for successful amplification. The successful PCR products were sent to Beijing Tsingke Biotechnology Co., Ltd (China) for sequencing of both strands using the original PCR primers. All sequences collected in this study have been submitted to GenBank and accession numbers are shown in Table 1.

### Molecular data analysis

The forward and reverse chromatograms were proofread and then assembled and edited using DNASTAR software (DNASTAR, Madison, Wisconsin, USA). Multiple sequence alignments were performed by CLUSTAL X 2.0.21 (Thompson et al. 1997; Jeanmougin et al. 1998). Primer sequences were manually deleted with BIOEDIT 7.0.9.0 (Hall 1999). To ensure that the correct target gene fragment was obtained, all sequences were checked in NCBI by Basic Local Alignment Search Tool (BLAST) (Altschul et al. 1990). To ensure nonexistence of stop codons and pseudogenes, the nucleotide sequences were translated to amino acids by MEGA 7 (Kumar et al. 2016). Sequence composition analyses were performed in MEGA 7. Pairwise genetic distances were calculated using the Kimura 2-parameter (K2P) model in MEGA 7 (Kimura 1980). Haplotypes were defined by DNASP 5.0 (Librado and Rozas 2009). The detailed statistics for haplotypes are shown in Table 1. The substitution saturation tests of 45 haplotype sequences segments were conducted in DAMBE 5.3.74 (Xia 2013) by comparing the index of substitution saturation (Iss) with critical values (Iss.c). To construct phylogenetic trees, neighbor joining (NJ), minimum evolution (ME), Bayesian inference (BI) and maximum likeihood (ML) analyses were performed. NJ and ME analyses (Saitou and Nei 1987) were performed in MEGA 7 under K2P substitution model. Branch support was measured using 1000 replicates in each analysis (Felsenstein 1985). Results were summarized as 50% majority consensus trees in MEGA 7. BI analysis was performed in MRBAYES 3.1.2 (Ronquist and Huelsenbeck 2003). The best-fit nucleotide evolution substitution model was selected by JMODELTEST 2.1.7 (Darriba et al. 2012). The Bayesian information criterion (BIC) was used to compare substitution models. The HKY+G model of nucleotide evolution was used. Two replicate runs with four independent Markov chain Monte Carlo (MCMC) chains (one cold chain and three hot chains) to conduct for 2 million generations, with trees sampled every 1000 generations with default parameter values. The average standard deviation of split frequency was lower than 0.01, indicating that the sampling of posterior distribution was adequate. The average standard deviation of split frequencies and Potential Scale Reduction Factor (PSRF) were used for examining convergence. The stationarity was determined in TRACER 1.5 (Rambaut and Drummond 2009) by plotting the log-likelihood values versus generation number and the effective sample sizes >200 for all parameters. After stationarity had been reached, the first 25% trees were discarded as burn-in and a 50% majority-rule consensus tree with the posterior probability considered as node support values was constructed by summarizing the remaining trees. ML analysis was performed in RAXMLGUI 1.3.1, a graphical front-end for RAXML (Silvestro and Michalak 2012). All ML analyses with thorough bootstrap were run 10 times starting from random seeds under the GTRGAMMA model. The bootstrap support value (BS) was evaluated by analysis with 1000 replicates. All tree topologies were displayed in FIGTREE 1.4 (Rambaut 2009).

## Results

### Morphological variation of *D. vulgaris*

Our specimens from China included representatives of both previously reported morphotypes of the aedeagus of *D. vulgaris*. They also exhibited a range of more subtle variation in the curvature of the aedeagal shaft in lateral view. Under the current morphology-based concept, this species can nevertheless be identified by the colour pattern and the presence of a shallow apical notch on the aedeagus in posterior view.

### Sequence composition

The COI sequences are 630bp in length after alignment and trimming. Details of nucleotide composition are listed in Table 2. As is typical for insect mtDNA, the gene is AT-rich (Liu et al. 2012).

**Table 2.** The average nucleotide composition of the COI sequences of *Deltocephalus*.

Group/Species	T (%)	C (%)	A (%)	G (%)	A+T (%)
YNA	32.8	18.8	34.0	14.1	56.8
YNB	33.1	18.3	33.5	15.1	66.6
ZJA	32.8	19.0	34.2	14.4	67.0
ZJB	32.9	18.9	34.1	14.1	67.0
FJA	33.0	18.3	33.8	14.9	66.8
FJB	33.0	18.4	34.1	14.5	67.1
HNA	33.1	18.4	33.9	14.6	67.0
HNB	33.0	18.3	34.0	14.7	67.0
GDB	33.0	18.3	34.0	14.7	67.0
GXA	33.0	18.4	33.9	14.7	66.9
GXB	33.0	18.3	33.9	14.8	66.9
A total of A	33.0	18.6	34.0	14.5	67.0
A total of B	33.0	18.4	33.9	14.7	66.9
A total of A and B	33.0	18.5	33.9	14.6	66.9
* D. pulicaris *	33.7	20.9	30.6	14.8	64.3
* D. uncinatus *	35.2	18.0	32.0	14.9	57.2

### Substitution saturation test

The results of haplotype sequences for the substitution saturation test indicate the value of Iss is smaller than Iss.c; namely, little substitutional saturation was detected, which is strongly informative for constructing phylogenetic trees.

### Analysis of the genetic distance and phylogenetic trees

**Figure 4.**
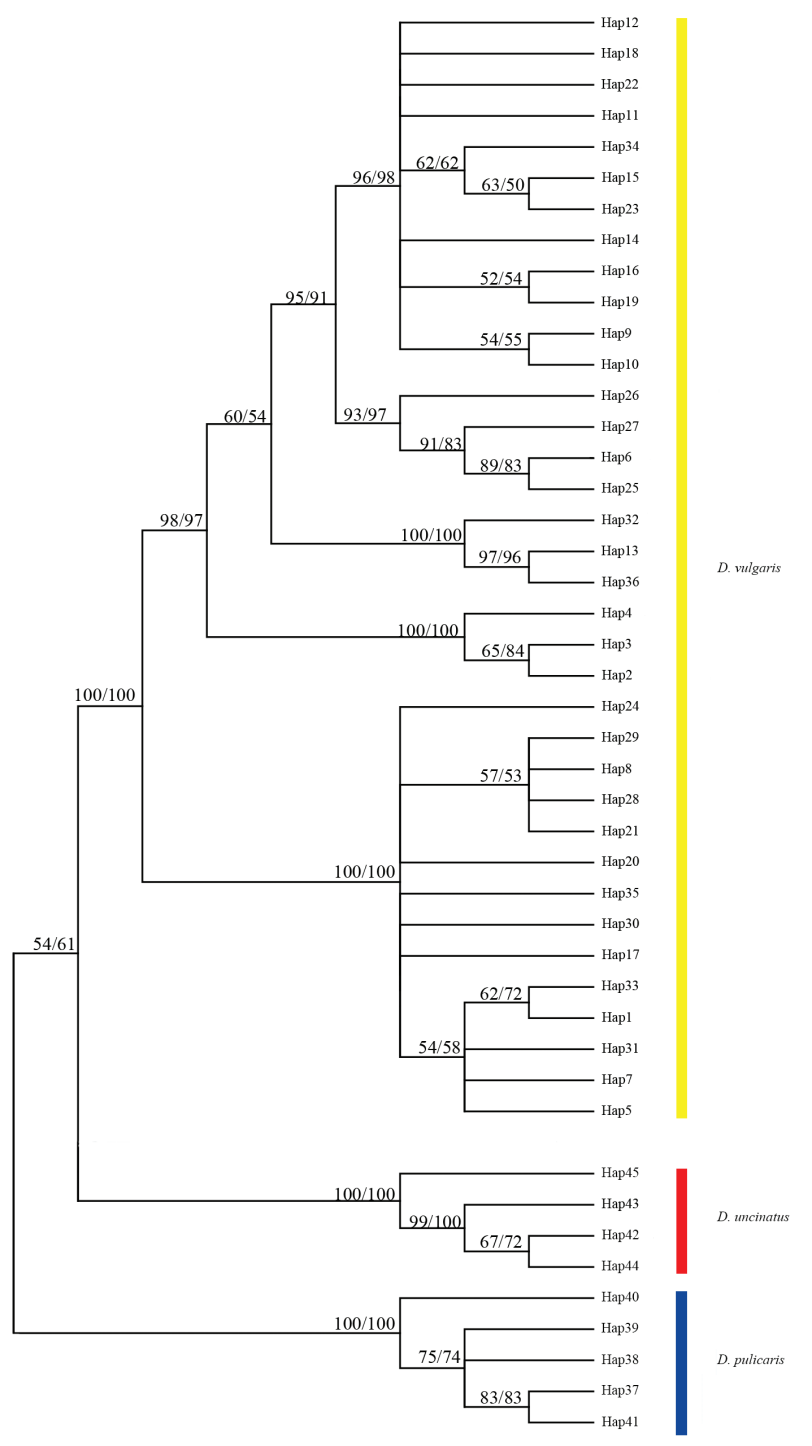
NJ/ME tree of 45 COI haplotypes. The node support: NJ/ME bootstrap values. Bootstrap values of less than 50 are not displayed.

**Figure 5.**
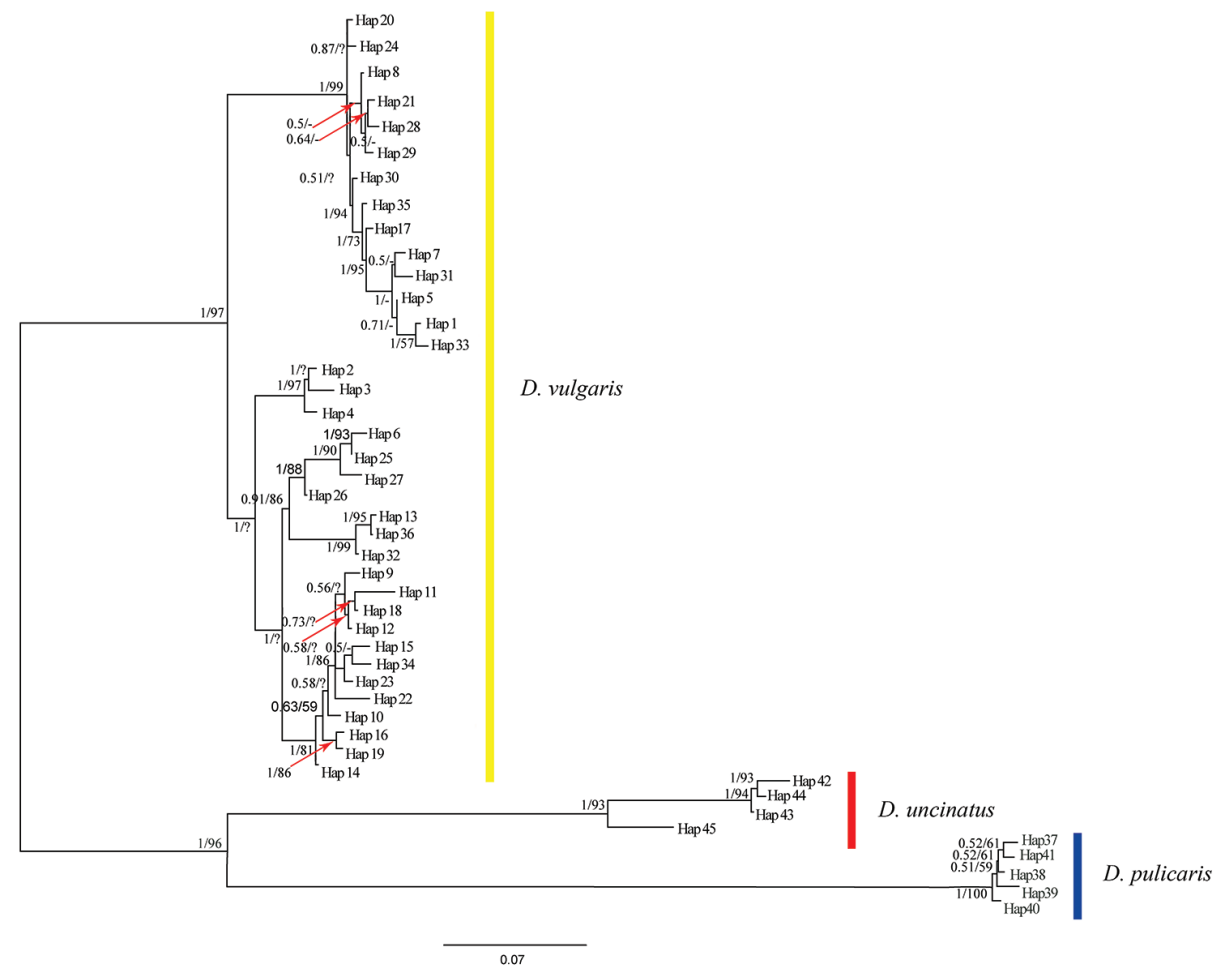
BI/ML tree of 45 COI haplotypes. The node support: BI posterior probabilities/ML bootstrap values. Posterior probabilities and bootstrap values under 0.5 and 50 are shown “-”. “?” means the positions of the different individual of *D. vulgaris* in ML tree is slightly different from those in BI tree.

The average genetic distances among morphological and geographical groups of *D. vulgaris* ranged from 0.9% to 6.3% and among species of *Deltocephalus* ranged from 16.4% to 21.9% without overlap (Table 3). This effectively reveals the existence of “DNA barcoding gap” and indicates the variation among morphological and geographical groups of *D. vulgaris* have not reached species level. Forty-five haplotypes (*D. vulgaris* – 36, *D. pulicaris* – 5, *D. uncinatus* – 4) were defined to perform phylogenetic analyses. The phylogenetic analyses based on NJ, ME, BI and ML methods nearly yielded identical trees except for the slight change of the position of a few individuals of *D. vulgaris* and bootsrap values (Figs 4, 5). *Deltocephalus vulgaris* haplotypes grouped into several distinct clades. However, these groups included individuals of both morphotypes and formed a distinct monophyletic clade with strong support value (BS(NJ) = 100, BS(ME) = 100, PP = 1, BS(ML) = 97) with no obvious biogeographic structure. Furthermore, different morphotypes of *D. vulgaris* share the same haplotype (Table 1). Thus, the COI sequence data suggest that previous authors were correct in treating the two morphotypes of *D. vulgaris* as belonging to the same species.

**Table 3.** Kimura 2-parameter genetic distances between groups/species of *Deltocephalus*.

	1	2	3	4	5	6	7	8	9	10	11	12
YNA												
YNB	0.047											
ZJA	0.041	0.063										
ZJB	0.041	0.057	0.017									
FJA	0.045	0.011	0.063	0.056								
FJB	0.043	0.029	0.047	0.043	0.023							
HNA	0.042	0.031	0.049	0.046	0.026	0.031						
HNB	0.044	0.014	0.062	0.056	0.007	0.022	0.023					
GDB	0.043	0.019	0.057	0.052	0.012	0.023	0.024	0.009				
GXA	0.045	0.023	0.050	0.046	0.021	0.030	0.032	0.021	0.024			
GXB	0.045	0.022	0.055	0.052	0.019	0.031	0.032	0.020	0.023	0.028		
* D. pulicaris *	0.207	0.219	0.206	0.204	0.212	0.206	0.210	0.210	0.209	0.212	0.213	
* D. uncinatus *	0.171	0.171	0.169	0.168	0.166	0.164	0.165	0.165	0.164	0.168	0.168	0.219

Note: the values indicate average intergroup and interspecific distances.

## Discussion

DNA barcoding as a standardised method to provide rapid and accurate species demarcation and has been widely applied in identifying and delimiting taxa since it was first reported by Hebert et al. (2003). Two standard criteria have generally been accepted in delimiting species using COI-based DNA barcodes. Based on the existence of a DNA barcoding gap, the feasibility of COI-based DNA barcoding depends on the fact that genetic distances among species are usually much higher than distances within species, without overlap. Different numbers of single species always form an independent clade in a phylogenetic tree (Wiens and Penkrot 2002; Hebert et al. 2003). Our analysis of COI sequences of *Deltocephalus* suggests a low level of genetic variation among morphotypes and geographical populations of *D. vulgaris*, and even different morphotypes of *D. vulgaris* share the same haplotype (e.g., YNA1 and YNB2; FJA1 and HNB1). The intergroup average genetic distances (0.9%–6.3%) of *D. vulgaris* among morphotypes and geographical populations is distinctly lower than that among species of *Deltocephalus* (16.4%–21.9%), without overlap. The phylogenetic tree (Figs 4, 5) recovered three independent lineages representing each of the three species with moderate to high support values. The genetic distances among a few morphotypes and geographical populations of *D. vulgaris* exceeded the 3% standard threshold (e.g., ZJA and HNB; YNA and HNA). The more detailed genetic distances between groups/species of *Deltocephalus* are summarized in Table 3. However, all individuals of *D. vulgaris* grouped into a single clade with strong support comprising several subordinate clades but with no obvious correspondence to morphological or geographic groups. Furthermore, different morphotypes from the same and different geographical distributions of *D. vulgaris* share the same haplotype (Table 1). We consider that the intraspecific genetic distance of a 3% standard threshold can be an inconsistent standard in different groups and maternal inheritance of mitochondrial genes can be affected in the process of evolution by the same mode of inheritance as Wolbachia infection, which also may result in a higher divergence in host mtDNA (Frezal and Leblois 2008; Muñoz et al. 2011). The low level of variation among morphotypes and geographical populations of *D. vulgaris* supports the notion that they represent a single species.

Differences in morphological characteristics, especially in male genitalia, have been the most reliable standard for discriminating among complex groups for many years. However, some cases of intraspecific variation in genital structures have been reported and these have led to uncertainty in the status of species and morphotypes. Mutanen et al. (2007) reported the male genital features that are most accepted and widely used standards to delimit species have been doubted in comparative study on male genital variation in *Pammene luedersiana* (Lepidoptera, Tortricidae). Yang et al. (2014) found 31 morphological variants in six species of *Mogannia* (Cicadidae, Cicadinae), but analysis of molecular data revealed low levels of intraspecific variation, although these morphological features have routinely been used to delimit species in this group. On the other hand, Wang et al. (2016) delimited seven different species of *Bundera* (Cicadellidae, Evacanthinae) based on molecular data, but only very minor morphological differences were found among six of these species. We are gradually becoming aware that similar morphological variation may have a different significance in different groups of leafhoppers and morphology-based species concepts may require confirmation using other kinds of data. DNA barcoding can efficiently complement morphology-based taxonomy and improve accuracy and rapidity in species identification.

*Deltocephalus vulgaris*, including 88 individuals in this study, and mainly representing two different forms of the aedeagus, were confirmed to be a single species grouped into a single clade with strongly support value in its phylogenetic trees (Figs 4, 5). Individuals collected both at the same place and time and different times and places have the same two forms of the aedeagus (e.g., FJA2 and FJB2; YNB10 and HNA2), which indicates forms are not related to temperature, humidity, precipitation, day length, altitude or latitude.

Our study shows a low intraspecific genetic distance between Guangdong and Hainan populations of *D. vulgaris* in southern China, suggesting that the Qiongzhou Strait (Fig. 1), a well-known biogeographic barrier has not significantly restricted gene flow for this species and they even share the same haplotype (Table 1). One logical assumption to explain this discovery is that Hainan and Guangdong arose earlier than the Qiongzhou Strait historically. Therefore, *D. vulgaris* freely exchanged genes when Guangdong and Hainan had been connected.

In the present study, lack of apparent correlation between morphology and COI haplotype is consistent with the hypothesis that the observed morphological variation is intraspecific. Nevertheless, we acknowledge the possibility that two different leafhopper species may share the same, or similar, COI haplotype. Thus, study of other genes may, in the future, reveal higher levels of divergence between the two forms and support recognition of some morphological variants as separate species.
